# Artificial Intelligence in Family Nursing: An Evidence‐Informed Policy Framework for Safe and Equitable Integration

**DOI:** 10.1111/inr.70237

**Published:** 2026-09-06

**Authors:** Honda Junko, Itoh Sakiko, Kubota Kazumi

**Affiliations:** ^1^ Research Institute of Nursing Care for People and Community University of Hyogo Hyogo Japan; ^2^ Graduate School of Health Care Sciences Institute of Science Tokyo Bunkyo Tokyo Japan; ^3^ Health Services Research and Development Center University of Tsukuba Tsukuba Japan; ^4^ Department of Women's Health Population Sciences National Center for Child Health and Development Setagaya Japan; ^5^ Department of Healthcare Information Management The University of Tokyo Hospital Bunkyo Tokyo Japan; ^6^ Shimonoseki City University Yamaguchi Japan

**Keywords:** artificial intelligence, digital health, family nursing, health equity, implementation, nursing policy

## Abstract

**Aim:**

To propose an evidence‐informed nursing policy framework for the safe, equitable and human‐centred integration of artificial intelligence into family nursing.

**Background:**

Ageing populations, chronic illness and the shift towards home‐ and community‐based care have increased reliance on families and family nursing. Artificial intelligence is increasingly used in healthcare for prediction, decision support, communication, remote monitoring and documentation. However, its implications for family nursing remain underexamined because family nursing involves relationships, shared decision‐making and family‐level information.

**Sources of Evidence:**

Academic and policy literature on artificial intelligence, digital health, family nursing, ethics, regulation, workforce development and implementation was reviewed in a structured manner and narratively synthesised to identify policy‐relevant opportunities, risks and requirements.

**Discussion:**

Artificial intelligence may help nurses identify risks earlier, tailor education and referrals, extend routine support through digital tools and evaluate family‐level outcomes. These opportunities must be balanced with concerns about privacy, consent, bias, digital exclusion, accountability and over‐reliance on automated recommendations. Japan is used as an illustrative context because rapid population ageing, strong family involvement and community‐based integrated care highlight issues relevant to many health systems.

**Conclusion:**

Safe integration requires five linked policy domains: ethical and data governance, workforce development, interdisciplinary co‐design, adaptive regulation and accountability, and sustainable funding and reimbursement.

**Implications for Nursing Practice:**

Nurses should use artificial intelligence–supported recommendations as prompts for professional assessment, not as replacements for relational judgement, family communication or human follow‐up.

**Implications for Nursing Policy:**

Policymakers, regulators, educators and health organisations should clarify accountability, protect family‐level data, embed artificial intelligence literacy in nursing education, require inclusive design and support reimbursement for nurse‐led, technology‐enabled family care.

## 1 Background

Family nursing is becoming increasingly important as populations age, chronic conditions become more common and care shifts from hospitals to homes and communities. Japan's rapidly ageing population provides a useful example of these pressures, but similar challenges are seen in many health systems that are trying to support older people and families outside hospital settings (Cabinet Office, Government of Japan 2024; Sakamoto et al. 2018). Japan's community‐based integrated care policy also illustrates how health systems are increasingly expected to connect medical, nursing, welfare and family support across local communities (Tsutsui 2014).

Family nursing focuses not only on the individual patient but also on family relationships, caregiving capacity, communication patterns, cultural expectations and the practical conditions in which care is provided. Foundational family nursing approaches emphasise that illness is experienced within family systems and that nurses need to assess both family strengths and family burden (Wright and Leahey 2013). Evidence from chronic illness contexts, such as parental involvement in adolescent type 1 diabetes care, also shows that family participation can influence health management and outcomes while requiring careful nursing support (Yamaguchi et al. 2023).

Family involvement can improve continuity and responsiveness of care, but it can also create strain. Family caregivers may experience uncertainty, emotional burden, financial pressure and difficulty navigating fragmented services. Family nursing addresses these issues by listening to family concerns, recognising hidden burdens, negotiating roles and helping families make care decisions that are realistic and acceptable. These relational and contextual dimensions make family nursing different from care models focused only on individual patients.

Artificial intelligence is now part of many healthcare policy and practice discussions. Current applications include predictive analytics, clinical decision support, conversational agents, remote monitoring, documentation support and risk stratification (Topol 2019). In nursing, artificial intelligence–based technologies may influence assessment, documentation, education, care coordination and patient engagement, although the evidence remains uneven across nursing domains (Buchanan et al. 2020; von Gerich et al. 2022). Nursing scholars and professional bodies have also argued that digital transformation should be shaped by nursing values, workforce preparation and nursing leadership rather than by technology alone (Booth et al. 2021; International Council of Nurses 2023).

The relevance of artificial intelligence to family nursing remains underexamined. Family nursing involves shared decision‐making, family‐level information and ongoing relationships. These features create policy questions that are not fully addressed by individual patient‐centred digital‐health models. International digital‐health guidance emphasises that digital technologies should strengthen health systems, improve equity and support health workers, but these goals require careful governance and context‐sensitive implementation (World Health Organization 2021b).

The introduction of artificial intelligence into family nursing may create new opportunities, but it also raises important risks. These include privacy and consent for family‐level data, algorithmic bias, digital exclusion, unclear accountability, over‐reliance on automated outputs and the possibility that technology may increase rather than reduce nurses’ workload. These concerns are particularly important when artificial intelligence is used with older adults, families with limited digital access or families experiencing social vulnerability.

A nursing policy framework is therefore needed to guide how artificial intelligence should be introduced into family nursing. Such a framework should clarify how family‐level data are protected, how nurses are prepared to use artificial intelligence, how families participate in design and evaluation, how accountability is assigned and how nurse‐led technology‐enabled care is funded. Without such policy guidance, artificial intelligence may be implemented unevenly and may fail to support the relational, ethical and practical work at the centre of family nursing.

## 2 Aim

This article aims to develop an evidence‐informed nursing policy framework for the safe, equitable and human‐centred integration of artificial intelligence into family nursing. Drawing on international academic and policy literature and using Japan as an illustrative context, it identifies policy‐relevant opportunities, risks and implementation requirements for policymakers, regulators, educators, health organisations and nursing leaders.

## 3 Sources of Evidence

This article is a non‐empirical, evidence‐informed policy analysis. It was not designed as a systematic review, meta‐analysis or formal evidence map. Instead, a structured search of academic and policy literature was conducted to identify key concepts, risks and implementation issues relevant to artificial intelligence in family nursing.

The search focused on literature available up to June 2025. PubMed, CINAHL and IEEE Xplore were searched using combinations of terms related to family nursing, family‐centred care, family caregiving, artificial intelligence, machine learning, digital health, nursing informatics, ethics, regulation, workforce development and implementation. International policy documents from major health and regulatory organisations were also reviewed when directly relevant to artificial intelligence, digital health, nursing practice or health‐system governance.

Sources were considered relevant when they addressed at least one of the following areas: artificial intelligence or digital health in nursing or healthcare; family nursing, family‐centred care or caregiver support; ethical, legal or governance issues in artificial intelligence; digital inclusion and workforce readiness; implementation, reimbursement or accountability issues related to health technologies. Recent international literature was prioritised, while foundational sources were retained when they provided concepts directly relevant to family nursing, technology adoption or nursing informatics.

Because artificial intelligence policy is rapidly evolving, a targeted update was conducted in July 2026 to identify major nursing, health and regulatory guidance published after June 2025. This update focused on major international and professional sources relevant to artificial intelligence governance, nursing leadership, health policy and regulation. Newly identified guidance, including a recent World Health Organization discussion of artificial intelligence in evidence‐informed health policy and a 2026 nursing position statement on artificial intelligence in health care, was reviewed to assess whether the proposed opportunity areas and policy domains required revision (American Academy of Nursing 2026; World Health Organization 2026). The update did not suggest the need to add or remove policy domains, and supported the continued relevance of the five domains, particularly the emphasis on human oversight, transparency, equity, workforce development, multidisciplinary governance and post‐implementation monitoring.

The selected sources, including those identified through the targeted update, were narratively synthesised to develop a policy argument rather than to estimate intervention effectiveness. The four opportunity areas and five policy domains were developed through an iterative synthesis by the three authors. First, the selected academic and policy sources were read to identify recurring issues relevant to artificial intelligence in family nursing, including clinical support functions, ethical risks, workforce readiness, implementation conditions, accountability and financing. These issues were then grouped into provisional categories and discussed among the authors. Alternative ways of organising the framework were considered, including by technology type, care setting and stakeholder group. However, the authors agreed that policy domains provided the clearest structure for identifying cross‐cutting implementation requirements.

The synthesis identified four policy‐relevant opportunity areas for artificial intelligence in family nursing: proactive risk identification, personalised intervention design, extended digital support and enhanced evaluation of family‐level outcomes. It also identified five policy domains required for safe and equitable integration: ethical and data governance, workforce development, interdisciplinary co‐design, adaptive regulation and accountability, and sustainable funding and reimbursement. The resulting framework is therefore an author‐proposed, evidence‐informed framework intended to guide discussion and local adaptation, rather than an empirically validated or internationally endorsed model.

## 4 Discussion

Artificial intelligence may support family nursing in several ways, but its value depends on how it is designed, governed, evaluated and integrated into nursing work. The following areas should therefore be understood as policy‐relevant opportunities proposed from the evidence‐informed synthesis, rather than as established effects specific to family nursing.

### 4.1 Proactive Risk Identification

Artificial intelligence may help nurses identify families who need earlier assessment or follow‐up. In healthcare, machine‐learning approaches have been used to support prediction, risk stratification and pattern recognition, but their usefulness depends on data quality, clinical context and careful interpretation (Rajkomar et al. 2019). In family nursing, similar approaches could potentially help identify caregiver burden, deteriorating health, social isolation, missed appointments or difficulties in self‐management.

Nursing data are important for this work because family needs, care coordination and patient responses are often documented through nursing practice. Brennan and Bakken (2015) argued that nursing must be visible in big data if health information systems are to reflect nursing contributions. However, predictive outputs should be treated as prompts for professional assessment, not as decisions. Algorithms can reproduce inequities when trained on biased or incomplete data, as shown in broader healthcare research on algorithmic bias (Obermeyer et al. 2019). For family nursing, this means that risk scores must be interpreted alongside family context, nurses’ judgement and the family's own concerns.

Outreach work by public health nurses with vulnerable pregnant women illustrates why human follow‐up remains essential. Even when systems help identify people who may need support, nurses still need to build trust, understand social vulnerability and respond to family circumstances that are not fully captured in data (Ishii and Honda 2025). Artificial intelligence may help signal need, but nurses remain essential in understanding what that need means in daily family life.

### 4.2 Personalised Intervention Design

Artificial intelligence may support the tailoring of education, referrals and care plans to family circumstances. Digital tools may help organise information about health literacy, caregiving roles, language needs, cultural preferences, available support and family concerns. This could assist nurses in selecting education materials, planning follow‐up or coordinating services.

Personalisation, however, should not be reduced to automated content delivery. Family nursing depends on conversation, trust and negotiation. Families may accept or reject support depending on whether they find it understandable, useful and compatible with their everyday lives. Technology acceptance research has long shown that perceived usefulness and ease of use influence whether people adopt new technologies (Davis 1989), and later models emphasised the importance of social, organisational and contextual factors in technology use (Venkatesh et al. 2003). These insights are relevant to family nursing because digital tools will not support families if they are burdensome, confusing or poorly fitted to care routines.

Digital literacy is also uneven among health professionals and communities. Research on health professionals’ digital literacy suggests that workforce readiness cannot be assumed and that digital‐health implementation requires training and organisational support (Tilahun et al. 2023). Policies should therefore require usability testing with nurses and families, culturally appropriate content, accessible language and mechanisms for correcting inaccurate or inappropriate recommendations.

### 4.3 Extended Digital Support

Conversational agents, remote monitoring and mobile applications may extend routine support between face‐to‐face encounters. These tools may provide reminders, basic education, symptom reporting or guidance on when to seek help. A recent quasi‐experimental study of an artificial intelligence–assisted breastfeeding counselling chatbot reported improvements in breastfeeding self‐efficacy and breastfeeding success at selected follow‐up points, although anxiety outcomes did not differ significantly between groups (Kerimoglu Yildiz et al. 2025). This study should be interpreted cautiously, but it suggests that digital tools may support some aspects of family‐related health education when embedded in a broader care system.

Such tools should not be presented as substitutes for nursing care. Families may need emotional support, practical problem‐solving and professional judgement that automated systems cannot provide. Guidance on large multi‐modal artificial intelligence models also highlights the need for careful governance, transparency, safety monitoring and attention to misinformation when generative tools are used in health contexts (World Health Organization 2025). In family nursing, policies should clarify the limits of digital tools, ensure that families know when and how to contact a nurse, and require safeguards for privacy, misinformation, crisis situations and digital exclusion.

The nursing profession also needs to be involved in shaping how these tools are introduced. The International Council of Nurses (2023) emphasises that nurses should participate in the design, implementation and evaluation of digital health technologies. This is particularly important in family nursing, where the quality of support depends on relationships, timing, trust and the practical realities of caregiving.

### 4.4 Enhanced Evaluation of Family‐Level Outcomes

Artificial intelligence may also support evaluation by helping organisations examine patterns in family‐level outcomes, care coordination, documentation and service use. Reviews of artificial intelligence–based technologies in nursing show that applications are expanding, but they also indicate that nursing‐specific outcomes and implementation contexts need stronger attention (von Gerich et al. 2022). Similarly, work on predicted influences of artificial intelligence across nursing domains suggests that artificial intelligence may affect clinical care, administration, education and research, but these changes require active nursing engagement (Buchanan et al. 2020).

Evaluation must include outcomes that matter to families and nurses. These may include caregiver burden, confidence, trust, continuity, timely follow‐up, workload and perceived usefulness. Policies should ensure that artificial intelligence–supported evaluation does not focus only on efficiency or cost. Family nursing requires measures that capture relational care, equity and the practical realities of caregiving.

Regulatory guidance also matters for evaluation. The World Health Organization's regulatory considerations for artificial intelligence in health emphasise the importance of risk management, transparency, performance evaluation and post‐deployment monitoring (World Health Organization 2023). These principles are directly relevant to family nursing because tools may affect not only individual patients but also family caregivers, care coordination and community support.

## 5 Policy Framework for Safe and Equitable Integration

The safe use of artificial intelligence in family nursing depends on more than whether a tool performs well technically. It depends on policy conditions that protect families, prepare nurses, clarify accountability and support human follow‐up. Based on the evidence‐informed synthesis described above, this analysis proposes five linked policy domains: ethical and data governance, workforce development, interdisciplinary co‐design, adaptive regulation and accountability, and sustainable funding and reimbursement.

Table 1 summarises the operational policy framework. It translates these domains into implementation challenges, recommended policy actions, possible indicators, contextual considerations and responsible stakeholders. The table is not intended to prescribe a single model of implementation. Rather, it is designed to help regulators, educators, health organisations and nursing leaders adapt artificial intelligence governance to local health systems, resources and family caregiving norms.

**TABLE 1:** Operational policy framework for artificial intelligence in family nursing.

Policy domain	Key implementation challenge	Recommended policy actions	Possible indicators	Contextual considerations	Responsible stakeholders
Ethical and data governance	Family nursing involves patient‐ and family‐level data, creating complex issues of consent, privacy and secondary use.	Establish family‐level data governance rules; require meaningful consent; use role‐based access; maintain audit trails; monitor bias and privacy incidents.	Existence of artificial intelligence governance policy; documented consent procedures; bias‐audit frequency; privacy incidents; family complaints and responses.	Expectations about family data sharing may differ by age, culture, care setting and legal context.	Regulators; health organisations; ethics committees; nursing leaders; data‐protection officers.
Workforce development	Nurses, students and family caregivers may have uneven confidence and competence in using artificial intelligence–supported tools.	Embed artificial intelligence literacy in nursing curricula and continuing education; train nurses to interpret uncertainty, recognise bias and explain limitations; provide family‐facing education materials.	Proportion of nurses completing training; inclusion of artificial intelligence competencies in curricula; availability of family education resources; nurse confidence and perceived usefulness.	Older caregivers, rural families and families with limited digital access may require low‐burden support.	Nursing schools; professional bodies; employers; community health agencies; nursing leaders.
Interdisciplinary co‐design	Tools may fail if they do not fit nursing workflow, family communication or cultural expectations.	Require co‐design with nurses, families, patients, data scientists, ethicists and policymakers; conduct usability and acceptability testing; revise tools after stakeholder feedback.	Evidence of stakeholder involvement; usability results; acceptability scores; workflow impact; documented changes after feedback.	Family roles, caregiving norms and communication preferences should shape interface design and escalation pathways.	Health organisations; vendors; nurses; families; data scientists; policymakers.
Adaptive regulation and accountability	Artificial intelligence systems may change over time, and responsibility for errors may be unclear.	Distinguish locked and adaptive systems; require post‐implementation monitoring; establish adverse‐event reporting; define human oversight and nurse override procedures.	Post‐market monitoring reports; adverse‐event mechanisms; documented human review; accountability pathways; audit records of overrides.	National laws, reimbursement systems, liability rules and digital infrastructure differ across countries.	Regulators; professional boards; health ministries; insurers; legal and risk‐management teams.
Sustainable funding and reimbursement	Preventive and relational nursing work supported by artificial intelligence may not be reimbursed.	Create payment models for nurse‐led remote monitoring, risk assessment, family education, digital counselling, care coordination and post‐alert follow‐up.	Reimbursement codes; implementation funding; cost‐effectiveness evidence; equity outcomes; caregiver outcomes; nurse workload measures.	Countries shifting care from hospitals to communities need funding models that recognise nurse‐led family support.	Health ministries; insurers; funders; health organisations; research agencies; nursing leadership.

*Note*: Artificial intelligence–supported recommendations should inform, not replace, nurses’ professional judgement. The proposed indicators are illustrative and have not been empirically validated. They require empirical testing and adaptation to local health systems, communities, workforce conditions, data‐protection requirements and regulatory arrangements.

Ethical and data governance is the foundation of artificial intelligence–supported family nursing. Family nursing often involves information about more than one person, including relationships, caregiving responsibilities and household circumstances. Policies should therefore address family‐level consent, role‐based access, data minimisation, audit trails, secondary use of data and bias monitoring. Ethical analyses of artificial intelligence in healthcare have repeatedly identified privacy, fairness, transparency and accountability as central concerns (Morley et al. 2020). These issues are especially important in family nursing because data may involve both patients and relatives.

Ethical implementation also requires attention to the clinical and organisational conditions in which artificial intelligence is used. Machine‐learning tools may appear objective, but their recommendations can be shaped by training data, institutional priorities and hidden assumptions. Char et al. (2018) emphasised that implementing machine learning in healthcare requires explicit attention to ethical challenges, including bias, responsibility and the changing relationship between clinicians and automated systems. For family nursing, these concerns mean that families should be informed when artificial intelligence is used and should have accessible ways to raise concerns.

Workforce development is also essential. Nurses need more than basic technical training. They need the ability to understand uncertainty, recognise potential bias, explain limitations to families and decide when human judgement should override artificial intelligence–supported suggestions. Artificial intelligence literacy should be embedded in undergraduate nursing education, continuing professional development and organisational training. This aligns with calls for the nursing profession to prepare for a digital future through education, leadership and participation in technology governance, and with recent nursing guidance emphasising that artificial intelligence should support rather than supplant nursing judgement (American Academy of Nursing 2026; Booth et al. 2021; International Council of Nurses 2023). Interdisciplinary co‐design is necessary because tools developed without nursing and family input may not fit real workflows or family needs. Nurses, families, patients, data scientists, ethicists, managers and policymakers should be involved from the early stages of design, testing and evaluation. Co‐design should include families with low digital access, older caregivers, rural communities and culturally diverse groups. This approach is consistent with international digital‐health strategy, which emphasises people‐centred, equitable and context‐sensitive digital transformation (World Health Organization 2021b).

Adaptive regulation and accountability are required because artificial intelligence systems may change over time and may be used differently across settings. Policies should distinguish between locked and adaptive systems, require post‐implementation monitoring, clarify adverse‐event reporting and define responsibility when artificial intelligence–supported recommendations contribute to harm. The World Health Organization's guidance on ethics and governance of artificial intelligence for health emphasises human autonomy, safety, transparency, responsibility, inclusiveness and sustainability (World Health Organization 2021a). A more recent World Health Organization discussion of artificial intelligence in evidence‐informed health policy also emphasises that artificial intelligence should augment, rather than replace, human judgement and should be governed through transparency, participatory engagement, multidisciplinary oversight and risk‐based approaches (World Health Organization 2026). The European Union Artificial Intelligence Act also illustrates the growing policy emphasis on risk management, transparency and accountability for high‐risk artificial intelligence systems (European Parliament and Council of the European Union 2024).

Sustainable funding and reimbursement are needed because many benefits of family nursing are preventive, relational and difficult to capture through conventional payment models. If reimbursement recognises only acute treatment or face‐to‐face encounters, nurse‐led digital follow‐up, family education, care coordination and post‐alert assessment may remain unsupported. Payment and funding models should therefore recognise the time and expertise required for safe technology‐enabled family care.

Japan provides an illustrative example of why these policy domains matter. Rapid population ageing, strong family involvement in care and community‐based integrated care create a setting where artificial intelligence may support coordination and earlier recognition of need (Cabinet Office, Government of Japan 2024; Tsutsui 2014). At the same time, Japan's health‐system structure, long‐term care arrangements and local service variation show why implementation must be adapted to system‐level realities (Sakamoto et al. 2018). These issues are not unique to Japan, but Japan provides a useful example of how demographic and system‐level pressures shape artificial intelligence policy needs in family nursing.

Across these domains, the central question is not whether artificial intelligence should be used in family nursing, but under what conditions it can be used safely, fairly and meaningfully. Artificial intelligence should strengthen nurses’ ability to support families, not narrow family nursing to automated risk scores or standardised messages.

## 6 Implications for Nursing Practice

For nursing practice, the main implication is that artificial intelligence should be used to strengthen relational care, not to reduce family nursing to automated tasks. Nurses should use artificial intelligence–supported recommendations as prompts for assessment and conversation. They should remain responsible for interpreting family context, recognising distress, negotiating care plans and advocating for families when technology does not fit their needs.

Nurses also need practical support to use these tools safely. This includes training in how to interpret uncertainty, recognise possible bias, explain limitations to families and decide when to question or override an automated recommendation. Families should be told when artificial intelligence–supported tools are being used, what the tools can and cannot do, and how to contact a nurse when they need human support.

Clinical and community settings should avoid digital‐only models of family support. Artificial intelligence may help organise information or signal possible risk, but many families need listening, reassurance, negotiation and practical problem‐solving. These elements remain central to family nursing and should be protected in any technology‐enabled model of care.

## 7 Implications for Nursing Policy

Several policy priorities follow from this analysis. First, artificial intelligence literacy should become part of nursing education and continuing professional development. Nurses need to understand what artificial intelligence can and cannot do, how bias may arise, how uncertainty should be communicated and when professional judgement should override an automated suggestion. Education should also prepare nurses to explain artificial intelligence–supported tools to families in clear, non‐technical language. This is consistent with international nursing and digital‐health guidance that emphasises workforce readiness, professional leadership and safe digital transformation (Booth et al. 2021; International Council of Nurses 2023; World Health Organization 2021b).

Second, health organisations should introduce artificial intelligence into family nursing through staged implementation and evaluation rather than rapid deployment. New tools should be tested with nurses and families before routine use. Evaluation should include safety, usability, workflow impact, family experience, equity, privacy and nurse workload. Organisations should also establish clear procedures for escalation, documentation and reporting concerns. Broader regulatory and ethical guidance supports this cautious approach, particularly for technologies that may influence clinical decisions or access to care (World Health Organization 2021a, 2023; European Parliament and Council of the European Union 2024).

Third, equity should be treated as a design and governance requirement. Artificial intelligence–supported family nursing may exclude families with limited digital access, low health literacy, language barriers or distrust of technology. Policies should require accessible formats, culturally appropriate communication, alternatives to digital‐only support and monitoring of unequal use or outcomes. Evidence on digital literacy among health professionals also suggests that implementation cannot rely on individual motivation alone; organisations must provide training, support and inclusive systems (Tilahun et al. 2023).

Fourth, reimbursement and funding policies should recognise nurse‐led technology‐enabled family care. Artificial intelligence may identify needs earlier, but benefits will not be realised unless nurses have time and resources to respond. Payment models should therefore support remote monitoring, digital counselling, family education, care coordination and post‐alert follow‐up as legitimate components of nursing care. This is particularly relevant in ageing societies and community‐based care systems, where family support and coordination are central to maintaining care at home (Cabinet Office, Government of Japan 2024; Sakamoto et al. 2018; Tsutsui 2014).

## 8 Limitations

This policy analysis has several limitations. First, although it was informed by a structured search of academic and policy literature, it was not designed as a systematic review and did not include exhaustive evidence mapping, formal quality appraisal or meta‐synthesis. The purpose was to develop an evidence‐informed policy framework rather than to estimate the effectiveness of specific artificial intelligence interventions.

Second, direct empirical evidence on artificial intelligence applications in family nursing remains limited. Some implications were therefore drawn from adjacent fields, including digital health, nursing informatics, community nursing, maternal and child health, ethics and health‐system regulation. These sources were used cautiously to identify policy issues rather than to claim established effectiveness in family nursing.

Third, Japan was used as an illustrative context, not as a universal model. Other countries differ in digital infrastructure, reimbursement, family caregiving norms, data‐protection laws and nursing roles. The framework should therefore be adapted to local systems and communities.

Finally, the framework has not yet been empirically evaluated. Future implementation research should examine how artificial intelligence–enabled family nursing tools affect family outcomes, caregiver burden, nurses’ workload, equity, privacy, cost and trust over time.

## 9 Conclusion

Artificial intelligence may support family nursing by helping nurses identify risks, tailor education, extend routine support and evaluate family‐level outcomes. These benefits, however, are not automatic. They depend on governance, workforce preparation, inclusive design, accountability and sustainable funding. This article proposes a five‐domain policy framework: ethical and data governance, workforce development, interdisciplinary co‐design, adaptive regulation and accountability, and sustainable funding and reimbursement. Artificial intelligence should remain accountable to nursing values and family needs. For policymakers and nursing leaders, the priority is to create conditions in which technology strengthens safety, equity, timeliness and human presence in family care.

## Author Contributions

Study concept and design: JH, SI and KK. Literature and policy source review: KK, SI and JH. Analysis and synthesis: JH, SI and KK. Manuscript writing (draft): KK. Critical revisions for important intellectual content: SI, JH and KK. Final approval of the manuscript: JH, SI and KK.

## Funding

The authors have nothing to report.

## Ethics Statement

Ethical approval was not required because this manuscript is a non‐empirical policy analysis and did not involve human participants, personal data collection, or access to identifiable records.

## Conflicts of Interest

The authors declare no conflicts of interest.

## Declaration on the Use of Artificial Intelligence–Assisted Tools

Artificial intelligence–assisted tools were used only for language editing, grammar checking and improving clarity of expression. They were not used to generate research questions, conduct analysis, develop the policy framework, interpret evidence or write conclusions. The authors reviewed and approved all content and take full responsibility for the accuracy and integrity of the manuscript.

## Data Availability

Data sharing is not applicable to this article because no datasets were generated or analysed.
